# 3-(4-Hy­droxy­phen­yl)-7-meth­oxy­chroman-4-one monohydrate

**DOI:** 10.1107/S1600536811046034

**Published:** 2011-11-05

**Authors:** Zhu-Ping Xiao, Zhu-Yun Peng, Qun Luo, Ying Wu, Ye-Ling Yang

**Affiliations:** a The Key Laboratory of Ecotourism Application Technology of Hunan Province and College of Chemistry and Chemical Engineering, Jishou University, Jishou 416000, People’s Republic of China

## Abstract

In the title compound, C_16_H_14_O_4_·H_2_O, the dihedral angle betwen the benzene rings is 71.4 (6)°. The pyran ring is in a sofa conformation. In the crystal, O—H⋯O hydrogen bonds connect the components into a two-dimensional network parallel to (010), incorporating *C*_2_^2^(4) and *C*_2_^2^(11) chains. In addition, weak C—H⋯O, C—H⋯π and π–π stacking inter­actions [centroid–centroid distance = 3.768 (2) Å] are present.

## Related literature

For background to and the biological activity of flavonoids, see: Xiao *et al.* (2007 ▶, 2010 ▶, 2011 ▶). For hydrogen-bond motifs, see: Bernstein *et al.* (1995 ▶). 
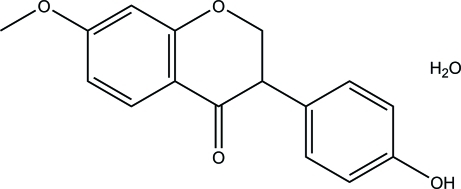


## Experimental

### 

#### Crystal data

C_16_H_14_O_4_·H_2_O
*M* *_r_* = 288.29
Monoclinic, 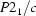

*a* = 9.730 (3) Å
*b* = 17.977 (5) Å
*c* = 8.570 (2) Å
β = 106.194 (2)°
*V* = 1439.6 (6) Å^3^
*Z* = 4
Mo *K*α radiation
μ = 0.10 mm^−1^
*T* = 296 K
0.30 × 0.20 × 0.20 mm

#### Data collection

Bruker SMART APEX CCD diffractometer
Absorption correction: multi-scan (*SADABS*; Sheldrick, 1996 ▶) *T*_min_ = 0.971, *T*_max_ = 0.981
11487 measured reflections
3113 independent reflections
2019 reflections with *I* > 2σ(*I*)
*R*_int_ = 0.031

#### Refinement

*R*[*F* ^2^ > 2σ(*F* ^2^)] = 0.051
*wR*(*F* ^2^) = 0.138
*S* = 1.04
3113 reflections
200 parameters
H atoms treated by a mixture of independent and constrained refinement
Δρ_max_ = 0.33 e Å^−3^
Δρ_min_ = −0.16 e Å^−3^

### 

Data collection: *SMART* (Bruker, 2007 ▶); cell refinement: *SAINT* (Bruker, 2007 ▶); data reduction: *SAINT*; program(s) used to solve structure: *SHELXS97* (Sheldrick, 2008 ▶); program(s) used to refine structure: *SHELXL97* (Sheldrick, 2008 ▶); molecular graphics: *SHELXTL* (Sheldrick, 2008 ▶); software used to prepare material for publication: *SHELXL97*.

## Supplementary Material

Crystal structure: contains datablock(s) global, I. DOI: 10.1107/S1600536811046034/lh5364sup1.cif

Structure factors: contains datablock(s) I. DOI: 10.1107/S1600536811046034/lh5364Isup2.hkl

Supplementary material file. DOI: 10.1107/S1600536811046034/lh5364Isup3.cml

Additional supplementary materials:  crystallographic information; 3D view; checkCIF report

## Figures and Tables

**Table 1:** Hydrogen-bond geometry (Å, °) *Cg*1 and *Cg*2 are the centroids of the C4–C9 and C10–C15 rings, respectively.

*D*—H⋯*A*	*D*—H	H⋯*A*	*D*⋯*A*	*D*—H⋯*A*
O4—H4⋯O5	0.82	1.78	2.585 (2)	167
O5—H5*A*⋯O2^i^	0.91 (3)	1.86 (3)	2.742 (3)	163 (3)
O5—H5*B*⋯O4^ii^	0.97 (5)	1.78 (5)	2.734 (3)	168 (4)
C8—H8⋯O5^iii^	0.93	2.55	3.397 (3)	152
C2—H2⋯*Cg*1^iv^	0.98	2.86	3.745 (3)	151
C6—H6⋯*Cg*2^v^	0.93	2.97	3.748 (3)	142

Symmetry codes: (i) 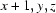; (ii) 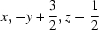; (iii) 
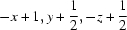
; (iv) 
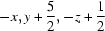
; (v) 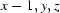.

**Fig. 1.**
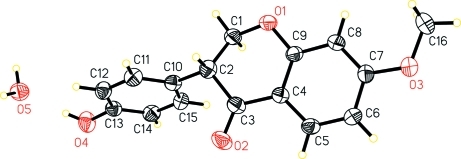
Molecular structure of the title compound. Displacement ellipsoids are drawn at the 30% probability level.

**Fig. 2.**
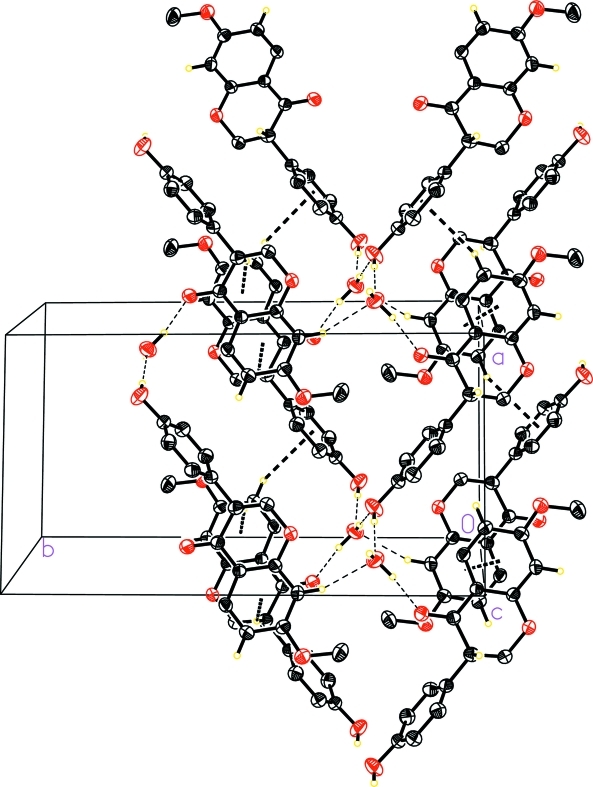
Part of the crystal structure with hydrogen bonds and other weak interactions shown as dashed lines. Only H atoms involved in hydrogen bonds are shown.

## supplementary crystallographic information

### Comment

Flavonoids are a large group of phenolic plant constituents. Approximately 9000 different flavonoids from different plant sources have been described so far, and each year, hundreds of newly identified flavonoids are being recorded in the literature (Xiao, *et al.*, 2011). Extensive epidemiological studies and *in vitro* experiments with polyphenols have indicate their broad variety of biological activities, including anticancer, anti-inflammatory, antibacterial, cardioprotective and enzyme-inhibitory activities (Xiao, *et al.*, 2007,2010).

The title compound crystallizes as a hydrate (Fig. 1). The C4-C9 ring forms a dihedral angle of 71.4 (6) ° with the C10-C15 ring. In the pyran ring, atoms C2-C4/C9/O1 are essentially planar with a mean deviation of 0.0115 Å and C1 is 0.535 (2) Å from the plane of these atoms.

In the crystal,  O—H···O hydrogen bonds connect the components into a two-dimensional network parallel to (010) incorparating C^2^_2_(4) and C^2^_2_(11) chains. In addtion, weak C—H···O, C—H···π(ring) interactions and π–π stacking interactions with a centroid to centroid distance of 3.768 (2) Å are present.

### Experimental

3-(4-hydroxyphenyl)-7-methoxy-4*H*-chromen-4-one (268 mg, 1 mmol) was dissolved in ethanol (10 ml). After 15 mg of 10% Pd/C was added under H_2_ atmosphere, the resulting mixture was stirred at room temperature for 6 h. After the catalyst was filtered off, the solvent was removed under reduced pressure. The residue was dissolved in ethanol (5 ml) and equal volume of water was then added. The crystals suitable for single crystal structure determination grown at room temperature by slow evaporation of a solution of the title compound in an ethanol and water mixture.

### Refinement

The H atoms bonded to O5 were located in difference Fourier maps and refined independently. All other H atoms were placed in geometrically idealized positions and constrained to ride on their parent atoms with C—H = 0.93 Å for aromatic H atoms, 0.96 Å for methyl H atoms, 0.97 Å for CH_2_, 0.98 Å for CH and 0.82 Å for the phenolic OH group. *U*_iso_(H) values were set at 1.2 times *U*_eq_(C) for aromatic C, the CH_2_ group and the CH group respectively, and 1.5 times *U*_eq_(O) or *U*_eq_(C) for phenolic OH group and the CH_3_ group.

### Crystal data

**Table:**

C_16_H_14_O_4_·H_2_O	*F*(000) = 608
*M**_r_* = 288.29	*D*_x_ = 1.330 Mg m^−^^3^
Monoclinic, *P*2_1_/*c*	Mo *K*α radiation, λ = 0.71073 Å
Hall symbol: -P 2ybc	Cell parameters from 2114 reflections
*a* = 9.730 (3) Å	θ = 2.7–26.4°
*b* = 17.977 (5) Å	µ = 0.10 mm^−^^1^
*c* = 8.570 (2) Å	*T* = 296 K
β = 106.194 (2)°	Block, colorless
*V* = 1439.6 (6) Å^3^	0.30 × 0.20 × 0.20 mm
*Z* = 4	

### Data collection

**Table:**

Bruker SMART APEX CCD diffractometer	3113 independent reflections
Radiation source: fine-focus sealed tube	2019 reflections with *I* > 2σ(*I*)
graphite	*R*_int_ = 0.031
φ and ω scans	θ_max_ = 27.0°, θ_min_ = 2.5°
Absorption correction: multi-scan (*SADABS*; Sheldrick, 1996)	*h* = −12→12
*T*_min_ = 0.971, *T*_max_ = 0.981	*k* = −22→22
11487 measured reflections	*l* = −10→10

### Refinement

**Table:**

Refinement on *F*^2^	Primary atom site location: structure-invariant direct methods
Least-squares matrix: full	Secondary atom site location: difference Fourier map
*R*[*F*^2^ > 2σ(*F*^2^)] = 0.051	Hydrogen site location: inferred from neighbouring sites
*wR*(*F*^2^) = 0.138	H atoms treated by a mixture of independent and constrained refinement
*S* = 1.04	*w* = 1/[σ^2^(*F*_o_^2^) + (0.0538*P*)^2^ + 0.3767*P*] where *P* = (*F*_o_^2^ + 2*F*_c_^2^)/3
3113 reflections	(Δ/σ)_max_ < 0.001
200 parameters	Δρ_max_ = 0.33 e Å^−^^3^
0 restraints	Δρ_min_ = −0.16 e Å^−^^3^

### Special details

**Table:**

Geometry. All e.s.d.'s (except the e.s.d. in the dihedral angle between two l.s. planes) are estimated using the full covariance matrix. The cell e.s.d.'s are taken into account individually in the estimation of e.s.d.'s in distances, angles and torsion angles; correlations between e.s.d.'s in cell parameters are only used when they are defined by crystal symmetry. An approximate (isotropic) treatment of cell e.s.d.'s is used for estimating e.s.d.'s involving l.s. planes.
Refinement. Refinement of *F*^2^ against ALL reflections. The weighted *R*-factor *wR* and goodness of fit *S* are based on *F*^2^, conventional *R*-factors *R* are based on *F*, with *F* set to zero for negative *F*^2^. The threshold expression of *F*^2^ > σ(*F*^2^) is used only for calculating *R*-factors(gt) *etc*. and is not relevant to the choice of reflections for refinement. *R*-factors based on *F*^2^ are statistically about twice as large as those based on *F*, and *R*- factors based on ALL data will be even larger.

### Fractional atomic coordinates and isotropic or equivalent isotropic displacement parameters (Å^2^)

**Table:**

	*x*	*y*	*z*	*U*_iso_*/*U*_eq_	
C1	0.2951 (2)	1.04367 (12)	0.2561 (3)	0.0630 (6)	
H1A	0.3289	1.0311	0.3705	0.076*	
H1B	0.3722	1.0691	0.2267	0.076*	
C2	0.2612 (2)	0.97338 (12)	0.1596 (3)	0.0559 (5)	
H2	0.2348	0.9874	0.0445	0.067*	
C3	0.1304 (2)	0.93648 (12)	0.1916 (3)	0.0541 (5)	
C4	0.03218 (19)	0.98545 (10)	0.2438 (2)	0.0454 (5)	
C5	−0.0930 (2)	0.95830 (11)	0.2750 (2)	0.0515 (5)	
H5	−0.1103	0.9074	0.2698	0.062*	
C6	−0.1897 (2)	1.00472 (11)	0.3125 (3)	0.0529 (5)	
H6	−0.2720	0.9855	0.3325	0.064*	
C7	−0.1647 (2)	1.08125 (12)	0.3209 (2)	0.0506 (5)	
C8	−0.0416 (2)	1.11027 (11)	0.2938 (3)	0.0540 (5)	
H8	−0.0246	1.1612	0.3000	0.065*	
C9	0.0559 (2)	1.06186 (11)	0.2570 (2)	0.0482 (5)	
C10	0.3882 (2)	0.92093 (11)	0.1881 (3)	0.0499 (5)	
C11	0.4480 (2)	0.90321 (12)	0.0668 (3)	0.0569 (5)	
H11	0.4123	0.9252	−0.0347	0.068*	
C12	0.5599 (2)	0.85359 (12)	0.0907 (3)	0.0568 (5)	
H12	0.5982	0.8421	0.0056	0.068*	
C13	0.6149 (2)	0.82111 (11)	0.2404 (3)	0.0523 (5)	
C14	0.5584 (2)	0.83907 (12)	0.3660 (3)	0.0587 (6)	
H14	0.5963	0.8181	0.4681	0.070*	
C15	0.4451 (2)	0.88844 (12)	0.3398 (3)	0.0577 (6)	
H15	0.4066	0.9000	0.4246	0.069*	
C16	−0.2487 (3)	1.20180 (15)	0.3705 (4)	0.0983 (10)	
H16A	−0.1630	1.2133	0.4545	0.147*	
H16B	−0.3294	1.2240	0.3962	0.147*	
H16C	−0.2411	1.2211	0.2688	0.147*	
H5A	0.949 (3)	0.8105 (17)	0.083 (3)	0.093 (9)*	
H5B	0.832 (4)	0.764 (2)	−0.042 (6)	0.162 (16)*	
O1	0.17584 (15)	1.09344 (8)	0.2323 (2)	0.0641 (4)	
O2	0.10853 (16)	0.87006 (9)	0.1665 (2)	0.0805 (6)	
O3	−0.26743 (16)	1.12282 (8)	0.3586 (2)	0.0687 (5)	
O4	0.72298 (18)	0.76992 (10)	0.2689 (2)	0.0773 (5)	
H4	0.7609	0.7711	0.1946	0.116*	
O5	0.8803 (2)	0.77481 (10)	0.0708 (3)	0.0741 (5)	

### Atomic displacement parameters (Å^2^)

**Table:**

	*U*^11^	*U*^22^	*U*^33^	*U*^12^	*U*^13^	*U*^23^
C1	0.0498 (12)	0.0499 (12)	0.0940 (18)	−0.0034 (10)	0.0277 (12)	−0.0004 (11)
C2	0.0477 (11)	0.0605 (13)	0.0574 (13)	0.0027 (10)	0.0113 (10)	0.0015 (10)
C3	0.0419 (11)	0.0472 (12)	0.0660 (14)	−0.0005 (9)	0.0032 (10)	−0.0042 (10)
C4	0.0413 (10)	0.0439 (11)	0.0468 (11)	−0.0009 (8)	0.0053 (9)	0.0008 (8)
C5	0.0479 (11)	0.0453 (11)	0.0573 (13)	−0.0062 (9)	0.0080 (10)	−0.0004 (9)
C6	0.0494 (11)	0.0544 (12)	0.0562 (13)	−0.0063 (9)	0.0166 (10)	0.0014 (10)
C7	0.0499 (11)	0.0540 (12)	0.0492 (12)	0.0061 (9)	0.0160 (9)	0.0016 (9)
C8	0.0583 (12)	0.0409 (11)	0.0648 (14)	0.0025 (9)	0.0206 (11)	0.0047 (9)
C9	0.0451 (11)	0.0453 (11)	0.0539 (12)	−0.0021 (9)	0.0133 (9)	0.0054 (9)
C10	0.0406 (10)	0.0476 (11)	0.0582 (13)	−0.0005 (9)	0.0083 (9)	0.0035 (10)
C11	0.0540 (12)	0.0622 (14)	0.0508 (13)	0.0036 (10)	0.0086 (10)	0.0074 (10)
C12	0.0581 (13)	0.0614 (13)	0.0520 (13)	0.0040 (10)	0.0170 (10)	0.0025 (10)
C13	0.0458 (11)	0.0469 (11)	0.0625 (14)	0.0035 (9)	0.0123 (10)	0.0042 (10)
C14	0.0630 (13)	0.0591 (13)	0.0521 (13)	0.0042 (11)	0.0128 (11)	0.0113 (10)
C15	0.0571 (12)	0.0606 (13)	0.0607 (14)	0.0006 (10)	0.0252 (11)	−0.0004 (11)
C16	0.102 (2)	0.0595 (16)	0.152 (3)	0.0176 (15)	0.067 (2)	−0.0024 (17)
O1	0.0566 (9)	0.0436 (8)	0.0990 (12)	−0.0018 (7)	0.0331 (8)	0.0061 (8)
O2	0.0518 (9)	0.0526 (10)	0.1353 (16)	−0.0072 (7)	0.0228 (10)	−0.0261 (10)
O3	0.0678 (10)	0.0585 (10)	0.0898 (12)	0.0046 (8)	0.0385 (9)	−0.0030 (8)
O4	0.0805 (11)	0.0754 (11)	0.0794 (12)	0.0351 (9)	0.0278 (9)	0.0175 (9)
O5	0.0637 (11)	0.0722 (12)	0.0863 (14)	−0.0155 (9)	0.0206 (10)	−0.0140 (10)

### Geometric parameters (Å, °)

**Table:**

C1—O1	1.434 (2)	C9—O1	1.367 (2)
C1—C2	1.496 (3)	C10—C11	1.363 (3)
C1—H1A	0.9700	C10—C15	1.392 (3)
C1—H1B	0.9700	C11—C12	1.378 (3)
C2—C10	1.519 (3)	C11—H11	0.9300
C2—C3	1.527 (3)	C12—C13	1.375 (3)
C2—H2	0.9800	C12—H12	0.9300
C3—O2	1.221 (2)	C13—O4	1.367 (2)
C3—C4	1.458 (3)	C13—C14	1.376 (3)
C4—C9	1.392 (3)	C14—C15	1.384 (3)
C4—C5	1.405 (3)	C14—H14	0.9300
C5—C6	1.362 (3)	C15—H15	0.9300
C5—H5	0.9300	C16—O3	1.432 (3)
C6—C7	1.395 (3)	C16—H16A	0.9600
C6—H6	0.9300	C16—H16B	0.9600
C7—O3	1.357 (2)	C16—H16C	0.9600
C7—C8	1.384 (3)	O4—H4	0.8200
C8—C9	1.386 (3)	O5—H5A	0.91 (3)
C8—H8	0.9300	O5—H5B	0.97 (5)
			
O1—C1—C2	113.81 (18)	O1—C9—C4	121.74 (17)
O1—C1—H1A	108.8	C8—C9—C4	121.99 (18)
C2—C1—H1A	108.8	C11—C10—C15	118.12 (19)
O1—C1—H1B	108.8	C11—C10—C2	121.57 (19)
C2—C1—H1B	108.8	C15—C10—C2	120.30 (19)
H1A—C1—H1B	107.7	C10—C11—C12	121.7 (2)
C1—C2—C10	113.06 (17)	C10—C11—H11	119.1
C1—C2—C3	109.50 (17)	C12—C11—H11	119.1
C10—C2—C3	112.51 (17)	C13—C12—C11	120.0 (2)
C1—C2—H2	107.1	C13—C12—H12	120.0
C10—C2—H2	107.1	C11—C12—H12	120.0
C3—C2—H2	107.1	O4—C13—C12	122.2 (2)
O2—C3—C4	123.18 (19)	O4—C13—C14	118.25 (19)
O2—C3—C2	120.41 (19)	C12—C13—C14	119.57 (19)
C4—C3—C2	116.32 (18)	C13—C14—C15	119.8 (2)
C9—C4—C5	117.36 (18)	C13—C14—H14	120.1
C9—C4—C3	120.86 (17)	C15—C14—H14	120.1
C5—C4—C3	121.71 (18)	C14—C15—C10	120.8 (2)
C6—C5—C4	121.66 (19)	C14—C15—H15	119.6
C6—C5—H5	119.2	C10—C15—H15	119.6
C4—C5—H5	119.2	O3—C16—H16A	109.5
C5—C6—C7	119.60 (19)	O3—C16—H16B	109.5
C5—C6—H6	120.2	H16A—C16—H16B	109.5
C7—C6—H6	120.2	O3—C16—H16C	109.5
O3—C7—C8	124.17 (19)	H16A—C16—H16C	109.5
O3—C7—C6	115.17 (18)	H16B—C16—H16C	109.5
C8—C7—C6	120.66 (18)	C9—O1—C1	114.27 (15)
C7—C8—C9	118.69 (18)	C7—O3—C16	118.38 (18)
C7—C8—H8	120.7	C13—O4—H4	109.5
C9—C8—H8	120.7	H5A—O5—H5B	113 (3)
O1—C9—C8	116.26 (17)		
			
O1—C1—C2—C10	−179.23 (17)	C5—C4—C9—C8	−2.2 (3)
O1—C1—C2—C3	−52.9 (2)	C3—C4—C9—C8	174.76 (19)
C1—C2—C3—O2	−158.3 (2)	C1—C2—C10—C11	−115.6 (2)
C10—C2—C3—O2	−31.6 (3)	C3—C2—C10—C11	119.7 (2)
C1—C2—C3—C4	25.1 (3)	C1—C2—C10—C15	65.6 (3)
C10—C2—C3—C4	151.69 (18)	C3—C2—C10—C15	−59.1 (3)
O2—C3—C4—C9	−174.3 (2)	C15—C10—C11—C12	1.2 (3)
C2—C3—C4—C9	2.3 (3)	C2—C10—C11—C12	−177.6 (2)
O2—C3—C4—C5	2.6 (3)	C10—C11—C12—C13	−0.6 (3)
C2—C3—C4—C5	179.13 (18)	C11—C12—C13—O4	178.0 (2)
C9—C4—C5—C6	1.7 (3)	C11—C12—C13—C14	−0.6 (3)
C3—C4—C5—C6	−175.30 (19)	O4—C13—C14—C15	−177.44 (19)
C4—C5—C6—C7	−0.1 (3)	C12—C13—C14—C15	1.3 (3)
C5—C6—C7—O3	179.76 (18)	C13—C14—C15—C10	−0.7 (3)
C5—C6—C7—C8	−0.9 (3)	C11—C10—C15—C14	−0.5 (3)
O3—C7—C8—C9	179.62 (19)	C2—C10—C15—C14	178.31 (19)
C6—C7—C8—C9	0.4 (3)	C8—C9—O1—C1	157.45 (19)
C7—C8—C9—O1	−179.64 (19)	C4—C9—O1—C1	−23.4 (3)
C7—C8—C9—C4	1.2 (3)	C2—C1—O1—C9	53.4 (3)
C5—C4—C9—O1	178.69 (18)	C8—C7—O3—C16	0.3 (3)
C3—C4—C9—O1	−4.3 (3)	C6—C7—O3—C16	179.6 (2)

### Hydrogen-bond geometry (Å, °)

**Table:**

Cg1 and Cg2 are the centroids of the C4–C9 and C10–C15 rings, respectively.

**Table:**

*D*—H···*A*	*D*—H	H···*A*	*D*···*A*	*D*—H···*A*
O4—H4···O5	0.82	1.78	2.585 (2)	167.
O5—H5A···O2^i^	0.91 (3)	1.86 (3)	2.742 (3)	163 (3)
O5—H5B···O4^ii^	0.97 (5)	1.78 (5)	2.734 (3)	168 (4)
C8—H8···O5^iii^	0.93	2.55	3.397 (3)	152.
C2—H2···Cg1^iv^	0.98	2.86	3.745 (3)	151
C6—H6···Cg2^v^	0.93	2.97	3.748 (3)	142

Symmetry codes: (i) *x*+1, *y*, *z*; (ii) *x*, −*y*+3/2, *z*−1/2; (iii) −*x*+1, *y*+1/2, −*z*+1/2; (iv) −*x*, *y*+5/2, −*z*+1/2; (v) *x*−1, *y*, *z*.
